# Effects of exposure to cigarette smoke prior to pregnancy in diabetic rats

**DOI:** 10.1186/1758-5996-3-20

**Published:** 2011-08-18

**Authors:** Débora C Damasceno, Yuri K Sinzato, Paula H Lima, Maricelma S de Souza, Kleber E Campos, Bruna Dallaqua, Iracema M Calderon, Marilza V Rudge, Gustavo T Volpato

**Affiliations:** 1 Botucatu Medical School, UNESP - Univ Estadual Paulista, Department of Gynecology and Obstetrics, Laboratory of Experimental Research in Gynecology and Obstetrics, São Paulo State, Brazil; 2 Pharmacology Department, University of Marilia_UNIMAR, Marilia, São Paulo State, Brazil; 3 Institute of Biological and Health Sciences, University Center of Araguaia, Mato Grosso Federal University (UFMT), Mato Grosso, Brazil

**Keywords:** rat, diabetes, cigarette smoke, pregnancy, reproductive outcome, oxidative stress

## Abstract

**Background:**

The purpose of this study was to evaluate the effects of cigarette smoke exposure before pregnancy on diabetic rats and their offspring development.

**Methods:**

Diabetes was induced by streptozotocin and cigarette smoke exposure was conducted by mainstream smoke generated by a mechanical smoking device and delivered into a chamber. Diabetic female Wistar rats were randomly distributed in four experimental groups (n minimum = 13/group): nondiabetic (ND) and diabetic rats exposed to filtered air (D), diabetic rats exposed to cigarette smoke prior to and into the pregnancy period (DS) and diabetic rats exposed to cigarette smoke prior to pregnancy period (DSPP). At day 21 of pregnancy, rats were killed for maternal biochemical determination and reproductive outcomes.

**Results:**

The association of diabetes and cigarette smoke in DSPP group caused altered glycemia at term, reduced number of implantation and live fetuses, decreased litter and maternal weight, increased pre and postimplantation loss rates, reduced triglyceride and VLDL-c concentrations, increased levels of thiol groups and MDA. Besides, these dams presented increased SOD and GSH-Px activities. However, the increased antioxidant status was not sufficient to prevent the lipid peroxidation observed in these animals.

**Conclusion:**

Despite the benefits stemming from smoking interruption during the pregnancy of diabetic rats, such improvement was insufficient to avoid metabolic alterations and provide an adequate intrauterine environment for embryofetal development. Therefore, these results suggest that it is necessary to cease smoking extensive time before planning pregnancy, since stopping smoking only when pregnancy is detected may not contribute effectively to fully adequate embryofetal development.

## Background

Diabetes mellitus (DM) is a group of diseases characterized by high blood glucose levels that result from defects in the body's ability to produce and/or use insulin [1]. In pregnancies complicated by diabetes, hyperglycemia and lipid metabolism alterations are associated with both maternal and fetal complications [2,3], causing reproductive abnormalities that enhance spontaneous abortion, congenital anomalies, and neonatal morbidity and mortality [4,5]. Oxidative stress has been implicated as a contributor to both the onset and the progression of diabetes and its associated complications. Some of the consequences of an oxidative environment are the development of insulin resistance, β-cell dysfunction, impaired glucose tolerance, and mitochondrial dysfunction, which can lead ultimately to the diabetic disease state. Experimental and clinical data suggest an inverse association between insulin sensitivity and ROS levels [6].

DM is a risk factor for atherosclerotic diseases, which can be aggravated by the presence of arterial hypertension, dyslipidemia and cigarette consumption [7]. It is a well-known fact that cigarette smoking constitutes a significant health hazard [8]. It has long been established that maternal smoking during pregnancy has adverse consequences for both the mother and the child [9,10]. The toxic effect of maternal exposure to tobacco smoke on the fetal metabolic status is indicated by the significant disturbance of the antioxidant status in newborns exposed *in utero *to tobacco smoke [11]. A significant negative correlation has been observed between the number of cigarettes smoked per day by pregnant women and newborns' weight, body mass index, length and head circumference [12].

Experimental diabetes induced by β-cytotoxic drug administration, such as streptozotocin, during the adult life of laboratory animals causes severe diabetic clinical status that reproduces uncontrolled human DM1. Severe diabetes (glycemia superior to 300 mg/dL) causes maternal hypertriglyceridemia and hypercholesterolemia, which compromises maternal-fetal metabolism during pregnancy in rats [13-15]. It causes increased maternal oxidative stress [14], increased rate of pre and post-implantation embryonic losses [16] and intrauterine growth restriction (IUGR) [17,18]. Diabetic rats exposed to cigarette smoke before and during pregnancy presented placentomegaly, increased placental index, small fetuses for pregnancy age [16] and decreased liver glycogen concentrations [19].

de Souza and colleagues [15] verified the association of severe diabetes and exposure to cigarette smoke into rat pregnancy was related to the incidence of hypertriglyceridemia, and this result was due to the diabetes and not to exposure to smoke. There was no alteration to protein metabolism at term pregnancy. Diabetes and cigarette smoke exposure led to the activation of the antioxidant system in an attempt to detoxify the organism in face of high lipid peroxidation, which can be characterized by the determination of reactive substances to thiobarbituric acid.

However, there are no reports concerning the association of diabetes and cigarette smoke exposure in rats that stopped being exposed to tobacco smoke in the pregnancy, aiming to reproduce the status of gravid women who stopped smoking when pregnancy is detected. Hence, the aim of this study was to evaluate the effects of such association on the maternal organism and on the development of offspring.

## Methods

### 1. Animals and experimental groups

Six-week-old female and nine-week-old male Wistar rats, weighing approximately 190 g and 220 g respectively, were obtained from Unesp - Universidade Estadual Paulista. During the two-week acclimatization and the experimental exposure periods, the rodents (5 rats per cage) were maintained in an experimental room under controlled conditions of temperature (22 ± 2°C), humidity (50 ± 10%), and a 12-hour light/dark cycle with *ad libitum *access to commercial diet (Purina^® ^rat chow Brazil) and tap water. Rats were randomly distributed into four groups (n minimum = 15/group): nondiabetic (ND) and diabetic rats exposed to filtered air (D), diabetic rats exposed to cigarette smoke prior to and into the pregnancy period (DS) and diabetic rats exposed to cigarette smoke prior to pregnancy period (pre-pregnancy) (DSPP). The Ethics Committee for Experimental Animal Research of the Botucatu School of Medicine/Unesp approved of the protocols used in this study.

### 2. Experimental design

**Figure 1:**
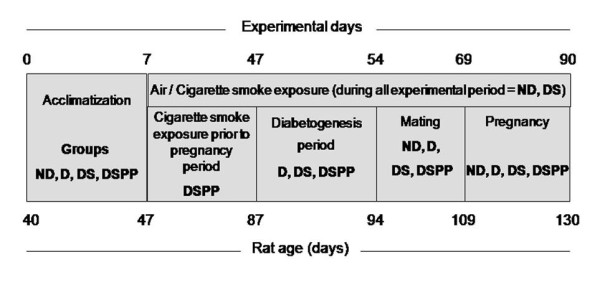
**Experimental design for association of the diabetes and mainstream cigarette smoke exposure**. **ND **= nondiabetic rats exposed to filtered air, **D **= diabetic rats exposed to filtered air; **DS **= diabetic rats exposed to cigarette smoke prior to and during pregnancy; **DSPP **= diabetic rats only exposed to cigarette smoke prior to pregnancy.

The experimental design (Figure 1) for association of the diabetes and cigarette smoke exposure in rats was modified by Lima and colleagues [20].

**Figure 2:**
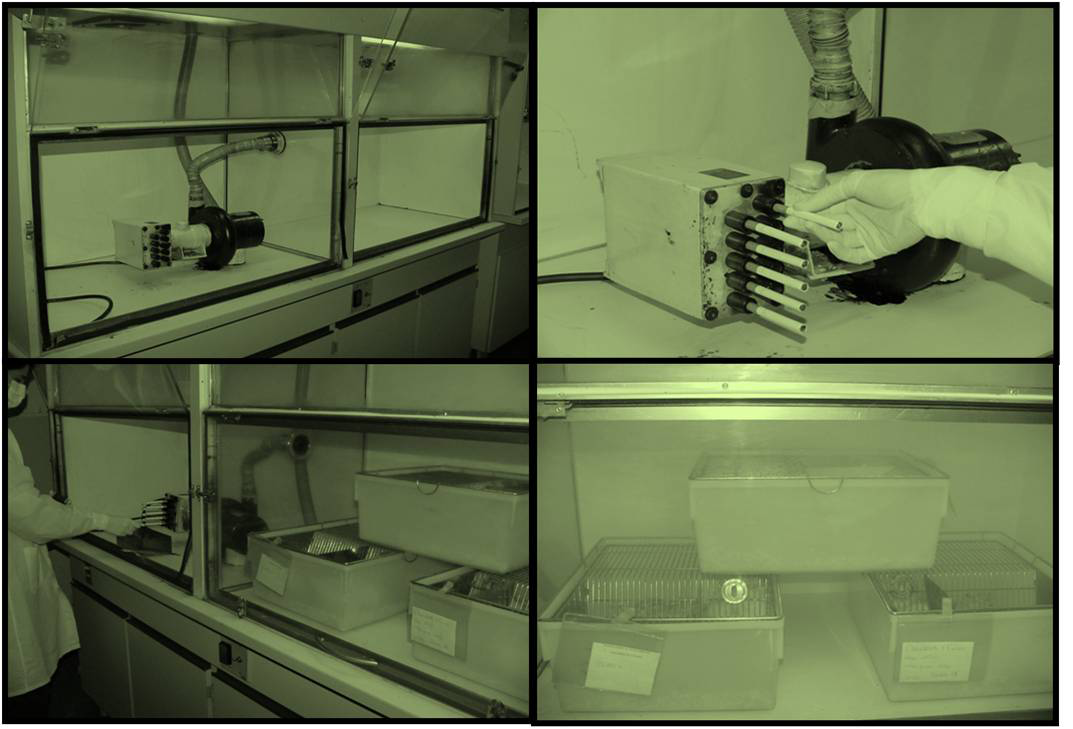
**Mainstream cigarette smoke apparatus in rats**.

A commercially available non-filter cigarette was used. Each cigarette contained 10 mg of tar, 0.80 mg of nicotine and 10 mg of carbon monoxide. Cigarette smoke exposure was conducted by mainstream smoke generated by a mechanical smoking device and delivered into a chamber (Figure 2). In the first moment (cigarette smoke adaptation period), non-pregnant rats were placed into whole-body exposure chambers to adapt to filtered air (control) or to cigarette smoke for 30 min (minutes), once a day (5 cigarettes/day), for 7 days. After adaptation to cigarette smoke exposure, the animals were placed into a chamber and exposed to smoke equivalent of 20 cigarettes/day for 30 min, 2 times each day, 7 consecutive days per week, for in turn 6 weeks. Each cigarette was puffed 15 times for 3 min at a rate of 5 puffs/min. Fresh air inhalation was performed for 1 min after every 3 min of cigarette smoke exposure. During the experiment, carbon monoxide was controlled in turn 195 ppm (particles/million), the temperature was maintained at 22-25°C, and relative humidity was approximately 40%. This protocol is similar to 3-4 pack/day in a human, considering the carboxyhemoglobin levels. The nondiabetic and diabetic rats exposed to filtered air were submitted to similar methodology but free of the cigarette smoke [21].

### 3. Induction of diabetes

Approximately six weeks after the beginning of the exposure to the air or cigarette smoke exposure, diabetes was induced by streptozotocin (STZ - SIGMA Chemical Company, St. Louis, Millstone). STZ was dissolved in citrate buffer (0.1 M, pH 6.5) and administered by intravenous (i.v.) injection at a dose of 40 mg/kg body weight (D, DS and DSPP). Non-diabetic (control) rats received (i.v.) only citrate buffer. During the induction of diabetes the animals are continuous being exposed to cigarette smoke/filtered air. For inclusion criteria, the diabetic dams showed glycemia superior to 300 mg/dL seven days after STZ injection, which is compatible with severe diabetes[22].

### 4. Mating Procedure

All female rats (ND, D, DS and DSPP) were mated overnight to nondiabetic male rats unexposed to cigarette smoke. The morning when sperm was found in the vaginal smear was designated gestational day 0. The mating procedure consisted of 15 consecutive days, a period comprising approximately three estral cycles, until a replicate number of groups were obtained. However, in this period non-mated female rats were considered to be infertile and discarded from the study. The DSPP group stopped to be exposed to cigarette smoke at day zero of pregnancy and the DS (cigarette smoke), ND and D (filtered air) groups ended this exposure at the 21st day of pregnancy.

### 5. Course of pregnancy

Glycemia was measured at days 0 and 21 of pregnancy in all experimental groups. At day 21 of pregnancy, the dams were weighed for estimation of body weight gain (maternal weight at day 21 minus maternal weight at day 0 of pregnancy) and were given an overdose of sodium pentobarbital (Hypnol^® ^3%) for laparotomy and collection of blood samples for biochemical determinations. The uterus was removed and weighed, and the ovaries and uterine contents examined to determine the number of corpora lutea and implantation sites, resorptions (embryonic death), and number and position of viable fetuses. The rate of embryonic loss before implantation was calculated as: (number of corpora lutea - number of implantations) × 100/number of corpora lutea, and used as a measure of failing conception effect or pre-implantation loss. The percentage of embryonic loss after implantation was calculated as: (number of implantations - number of live fetuses) × 100/number of implantations, which was used as a measure of abortifacient effect or for identification of post-implantation loss [23]. In the lack of visible implantation sites, the uterine corns were stained with a preparation of 10% ammonium sulphate [24]. Immediately after exploratory laparotomy, all viable fetuses and placentas were weighed for determination of the placental index (placental weight/fetal weight) [25].

### 6. Biochemical measurements

The blood samples were divided in two ways: one portion of blood was put into anticoagulant-free test tubes and in others containing anticoagulant. The blood samples without anticoagulant were kept at low temperature for 30 minutes (min) and then centrifuged at 1,300 × *g *for 10 min at 4°C. The supernatant was collected as serum and stored at -80°C for determination of total cholesterol (and fractions), triglycerides and proteins were determined by Wiener^® ^assay kit.

The blood samples were put into anticoagulant tubes, centrifuged at 900 × *g *for 10 min at room temperature for assay of oxidative stress biomarkers, which were estimated in the washed erythrocytes. The oxidative stress biomarkers evaluated were superoxide dismutase (SOD), thiol groups and malonaldehide (MDA). MDA was measured as the lipid peroxidation index. SOD enzymatic activity unit was defined as SOD units able to produce 50% of pyrogallol oxidation inhibition. All data were expressed in units of SOD per milligram of hemoglobin. Thiol groups was enzymatically determined using 5,5'-dithio-bis (2-nitrobenzoic acid) (DTNB) and glutathione reductase in the presence of a reduced form of nicotinamide adenine dinucleotide phosphate (NADPH), forming 2-nitro-5-thiobenzoic acid. Thiol group concentration was measured at 412 nm on a spectrophotometer. One unit of its activity was equal to the micromolar of substrate reduced per gram of hemoglobin. For MDA concentration, the absorbance was measured at a wavelength of 535 nm, and the results were expressed as nM of MDA per gram of hemoglobin (nM/g Hb) [15].

### 7. Statistical analysis

The glycemic levels and maternal reproductive outcomes were expressed as mean ± standard deviation, and other biochemical data were reported as mean ± standard error of mean. The results of glycemia, lipid and protein determination, oxidative stress status (for MDA and SOD), and maternal reproductive outcomes (for litter weight, maternal weight gain, fetal and placental weights) were analyzed by Tukey multiple comparison test [26]. When data presented no symmetrical distribution and homogenize variance, Gamma distribution was used (for pre- and postimplantation loss rates, GSH-Px and thiol groups). Poisson regression was applied for implantation sites, live fetuses, corpora lutea numbers. Statistical significance was considered as p < 0.05. All data were evaluated according to help of the statistical professional.

## Results

### 1. Maternal glycemia during pregnancy

**Figure 3:**
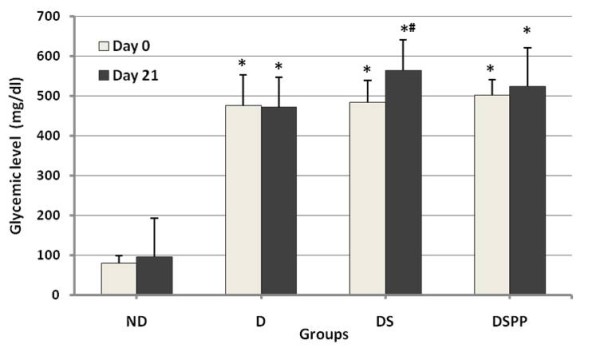
**Maternal glycemia (mg/dL) at days 0 and 21 of pregnancy in the different experimental groups**. Values are expressed as mean ± standard deviation (SD). ND = nondiabetic rats exposed to filtered air, D = diabetic rats exposed to filtered air; DS = diabetic rats exposed to cigarette smoke prior to and during pregnancy; DSPP = diabetic rats only exposed to cigarette smoke prior to pregnancy. * p < 0.05 - significant statistically difference as compared to ND group; ^# ^p < 0.05 - significant statistically difference as compared to D group (Tukey multiple comparison test).

The diabetic rats (D, DS and DSPP) presented increased glycemia (p < 0.05) in relation with nondiabetic rats at beginning and at term pregnancy. The rats exposed to cigarette smoke prior to and into the pregnancy period (DS) presented increased levels of glycemia only at day 21 of pregnancy as compared to D group (Figure 3) (p < 0.05).

### 2. Maternal reproductive outcomes

In DS and DSPP dams, there was reduced number of implantation (8.25 ± 3.50 and 7.57 ± 3.57), live fetus number (7.25 ± 3.41 and 6.36 ± 4.27), litter weight (49.63 ± 18.07 and 46.05 ± 20.89) and maternal weight gain (73.31 ± 21.24 and 46.58 ± 48.61); and increase (p < 0.05) in preimplantation loss rate (32.43% and 33.69%) in relation to ND (implantation = 12.63 ± 0.80; live fetuses = 12.18 ± 0.90; litter weight = 84.21 ± 6.98; maternal weight gain = 133.09 ± 14.99; preimplantation loss rate = 5.97%) and D (12.00 ± 1.26; 10.90 ± 0.70; 73.31 ± 4.99; 107.38 ± 12.07; 6.36%) groups, respectively. The diabetic rats (D, DS and DSPP) presented decreased fetal weight (approximately 4.4 ± 0.5) and increased placental weight (approximately 0.62 ± 0.10) (p < 0.05) as compared to ND group (5.31 ± 0.29; 0.48 ± 0.03), respectively. The rates of postimplantation loss in DS and DSPP (13.26% and 27.85%) were higher in relation with ND group (2.72%), and this rate was increased in DSPP (27.85%) as compared to D group (6.35%) (Table 1).

**Table 1:** Maternal reproductive outcome evaluated at day 21 of pregnancy from different experimental groups.

Variables	Groups			
	
	ND (n = 15)	D (n = 15)	DS (n = 15)	DSPP (n = 17)
**No. of corpora lutea^a^**	13.54 ± 1.03	12.81 ± 1.72	12.08 ± 1.62	11.71 ± 2.84
**No. of implantation sites^a^**	12.63 ± 0.80	12.00 ± 1.26	8.25 ± 3.50*^#^	7.57 ± 3.57*^#^
**No. of live fetuses^a^**	12.18 ± 0.90	10.90 ± 0.70	7.25 ± 3.41*^#^	6.36 ± 4.27*^#^
**Litter weight^b^**	84.21 ± 6.98	73.31 ± 4.99	49.63 ± 18.07*^#^	46.05 ± 20.89*^#^
**Maternal Weight Gain^b^**	133.09 ± 14.99	107.38 ± 12.07	73.31 ± 21.24*^#^	46.58 ± 48.61*^#^
**Fetal weight^b^**	5.31 ± 0.29	4.41 ± 0.52*	4.49 ± 0.30*	4.23 ± 0.85*
**Placental weight^b^**	0.48 ± 0.03	0.61 ± 0.07*	0.62 ± 0.13*	0.66 ± 0.29*
**Preimplantation loss (%)^c^**	5.97	6.36	32.43*^#^	33.69*^#^
**Postimplantation loss (%)^c^**	2.72	6.35	13.26*	27.85*^#^

Values are expressed as mean ± standard deviation (SD).

ND = nondiabetic rats exposed to filtered air, D = diabetic rats exposed to filtered air; DS = diabetic rats exposed to cigarette smoke prior to and during pregnancy; DSPP = diabetic rats only exposed to cigarette smoke prior to pregnancy.

* p < 0.05 - significant statistically difference as compared to ND group; ^#^p < 0.05 - significant statistically difference as compared to D group. ^a^Zero - Inflated Poisson; ^b^Normal distribution (Tukey Multiple Comparison test), and ^c^Gamma distribution.

### 3. Biochemical measurements

The biochemical parameters evaluated at day 21 of pregnancy are presented in Table 2. The D rats presented increase in VLDL-c, triglycerides, total cholesterol and MDA levels as compared to ND group. The DS dams showed increased determinations of HDL-c, VLDL-c, triglycerides, MDA, and activities of GSH-Px and SOD in relation with ND group; besides HDL-c level and GSH-Px activity were also increased as compared to values from D group. The DSPP dams exhibited significant statistically increase in MDA and thiol group levels, SOD and GSH-Px activities in relation with ND rats. As compared to D group, the DSPP rats presented reduction in the VLDL-c and triglycerides levels and increase in the thiol group levels and SOD activity. DSPP also showed an increased thiol group measurement as compared to level from DS group.

**Table 2:** Biochemical measurements evaluated at day 21 of pregnancy from different experimental groups.

Variables	Groups			
	
	ND (n = 15)	D (n = 15)	DS (n = 15)	DSPP (n = 17)
**HDL-c** **(mg/dL)^a^**	32.21 ± 7.88	43.84 ± 4.71	94.86 ± 9.31*^#^	66.10 ± 7.65
**VLDL-c** **(mg/dL)^a^**	66.23 ± 5.35	304.63 ± 33.24*	206.91 ± 23.63*	170.90 ± 27.79^#^
**Triglycerides (mg/dL)^a^**	331.16 ± 26.81	1523.16 ± 166.22*	1034.53 ± 118.16*	854.61 ± 138.97^#^
**Total cholesterol (mg/dL)^a^**	118.15 ± 5.97	287.57 ± 38.01*	226.60 ± 21.99	238.22 ± 33.65
**Total protein (g/dL)^a^**	6.73 ± 0.7714	7.30 ± 0.53	5.60 ± 0.56	7.08 ± 0.80
**MDA** **(nM/g Hb)^a^**	49.85 ± 4.18	686.84 ± 298.35*	451.14 ± 119.61*	696.03 ± 86.37*
**SOD** **(U/mg Hb)^a^**	1.78 ± 0.30	11.64 ± 4.23	17.48 ± 2.59*	22.19 ± 1.44*^#^
**GSH-Px** (**U/mg Hb)^b^**	0.08 ± 0.03	0.35 ± 0.15	1.83 ± 0.53*^#^	1.60 ± 0.15*
**Thiol groups (μM/g Hb)^b^**	1.19 ± 0.47	3.81 ± 0.95	5.52 ± 0.44	24.54 ± 5.23*^#$^

Values are expressed as mean ± standard error of mean (SEM).

ND = nondiabetic rats exposed to filtered air, D = diabetic rats exposed to filtered air; DS = diabetic rats exposed to cigarette smoke prior to and during pregnancy; DSPP = diabetic rats only exposed to cigarette smoke prior to pregnancy.

*p < 0.05 - significant statistically difference as compared to ND group; ^#^p < 0.05 - significant statistically difference as compared to D group; ^$^p < 0.05 - significant statistically difference as compared to DS group. ^a^Normal distribution (Tukey Multiple Comparison test) and ^b^Gamma distribution.

## Discussion

Cigarette smoke exposure did not significantly exacerbate the hyperglycemia in diabetic groups at day 0 of pregnancy, corroborating with other investigations performed in our laboratory, which verified that rats exposed to cigarette smoke prior to and during pregnancy presented glycemia similar to diabetic dams [15,20]. However, our study showed an increased glycemia in the end of pregnancy in diabetic rats exposed to cigarette smoke prior to and the pregnancy period.

The analysis of maternal reproductive performance was aggravated in the diabetes/cigarette smoke association, regardless of the exposure moments. The number of implanted embryos and of live fetuses that developed was lower in the groups of rats submitted to the association of two variables, thus contributing to reduction of the litter weight. de Souza et al [16] also observed these alterations in the diabetic rats exposed to cigarette smoke prior to and into pregnancy period. The embryonic loss rate prior to implantation depends on the number of corpora lutea and implantation sites. In this study, it was observed that the number of implantation sites was smaller in groups DS and DSPP, leading to increased percentages of preimplantation loss in the respective groups. For an embryonic fixation (embryonic implantation) to occur in the gravid endometrium there must be a synchronism between the maternal organism and embryonic development. The cigarette contains high levels of toxic substances that may accumulate in reproductive organs, thus contributing to impair the embryonic implantation process. By this way, studies indicates that smoking one pack of cigarettes per day and starting to smoke before 18 years of age is associated with an increased risk of infertility in females [27]. The rats that were submitted to cigarette smoke exposure prior to pregnancy period (DSPP) showed fetal viability reduction, similarly to those which continued being exposed during the whole experiment (DS). The post implantation loss rates were increased after the diabetes/tobacco smoke association, but these results were aggravated when the rats abruptly stopped being exposed to cigarette smoke, suggesting an increased inflammatory reaction due to elevation of cortisol caused by abstinence syndrome.

In the present study, cigarette smoke exposure prior to pregnancy did not improve reduction of fetal weights from diabetic dams exposed to cigarette smoke before and during pregnancy, showing that stop to being exposed previous moments of pregnancy is not enough. It is known that decreased weight at birth presents a direct relation between the number of cigarettes smoked, carbon-monoxide concentration and cigarette tar content. However, despite the numerous investigations, the mechanism by which smoking interferes with fetal development remains unclear. The offspring of women who smoked during pregnancy are born with tobacco combustion products in their organisms which are harmful to their development. Such newborns usually present lower birth weight, are less developed and more vulnerable to diseases, including asthma and even cancer [28]. Data have shown an association between abnormal fetal growth and both active [29] or passive [30] cigarette smoke exposure during gestation. Esposito and colleagues [31] identified a temporal window of vulnerability during the pre/peri-implantation period of embryonic development for *in utero *smoke-exposure-induced low birth weight. Further, these studies documented the establishment of a viable animal model with which to test hypotheses regarding the cellular and molecular mechanisms underlying gestational low birth weight induced by tobacco-smoke exposure.

Previous study performed in our laboratory verified that diabetic rats (D) presented high levels of triglycerides and total cholesterol [15]. In our paper, the rats that stopped smoking prior to pregnancy period (DSPP) showed decreased triglyceride levels, and consequently reduction in the VLDL-c levels. However, the reduced level of this lipid was not sufficient to characterize a total improved lipid profile in these animals. Besides, the total cholesterol tended to increase in diabetic dams and diabetes and cigarette association group. These animals also presented high level of MDA, thus elevated lipoperoxidation (exacerbated oxidative stress status), contributing to an adverse intrauterine environment, which led to an impaired embryofetal development (reduced number of implantations and live fetuses). In our paper, the rats that stopped smoking prior to pregnancy period (DSPP) showed a tendency to increase SOD activity and significant increase in levels of thiol groups. We suggested that the abstinence syndrome status caused metabolism alterations, which exacerbated oxidative stress, but the increase of antioxidants (thiol groups) status was not sufficient to prevent the lipid peroxidation observed in these animals. In physiologic concentrations, ROS help to maintain homeostasis. However, when ROS accumulate in excess for prolonged periods of time, they cause chronic oxidative stress and adverse effects [32].

This data confirmed that stopping smoking prior to pregnancy period presented similar data as compared to be submitted to cigarette smoke before and during pregnancy. In conclusion, despite the benefits stemming from smoking interruption during the pregnancy of diabetic rats, such improvement was insufficient to avoid metabolic alterations and provide an adequate intrauterine environment for embryofetal development. Therefore, these results suggest that it is necessary to cease smoking extensive time before planning pregnancy since stopping smoking only when pregnancy is detected may not contribute effectively to fully adequate embryofetal development.

## Conflict of interests

The authors declare that they have no competing interests.

## Authors' contributions

All authors have participated in the manuscript's design and drafting. All authors have participated in the study's review of the data shown and they have read and approved of the final manuscript version.
